# Should Complex Cancer Patients Requiring High-Risk Surgery Shoot for the Stars?

**DOI:** 10.1093/jncics/pkaa061

**Published:** 2020-07-07

**Authors:** Fahima Dossa, Nancy N Baxter

**Affiliations:** p1 Division of General Surgery, Department of Surgery, University of Toronto, Toronto, ON, Canada; p2 Institute of Health Policy, Management, and Evaluation, University of Toronto, Toronto, ON, Canada; p3 Melbourne School of Population and Global Health, University of Melbourne, Melbourne, Victoria, Australia

To provide patients with understandable, accessible, hospital-quality metrics, the US Centers for Medicare and Medicaid Services (CMS) developed the Overall Hospital Quality Star Ratings. This system amalgamates data from various Hospital Compare measures, grouping measures into 7 weighted categories reflecting patient outcomes (mortality, safety, readmission), patient experience, processes of care (effectiveness, timeliness), and efficiency of care (efficient use of medical imaging). Composite scores from each category are used to generate a summary score, which is translated into a star rating from 1 to 5, reflecting hospital performance (1). CMS Star Ratings are recommended as a “starting point” to compare hospitals in nonemergency situations (2).

Cancer care can be complex and surgical management carries substantial risk; the CMS Star Rating presents an opportunity for cancer patients to select hospitals where quality of care may be higher and surgical risk lower. An association between CMS Star Rating and mortality after cancer surgery has previously been demonstrated (3), suggesting if all patients selected high-ranked hospitals, postoperative mortality could be reduced.

Using Medicare data for patients undergoing 1 of 5 high-risk complex cancer surgeries, Papageorge et al. (4) report higher 90-day mortality at 1-star compared with 5-star hospitals (10.4% vs 6.4%); differences were greatest for esophagectomy (19.2% vs 11%) and pancreaticoduodenectomy (17.1% vs 8.1%). The authors then modeled a scenario where all patients undergoing these 5 surgeries at 1-star hospitals were instead treated at 5-star hospitals and found this would reduce 90-day mortality from 10.4% to 6.6%. Relocation of these Medicare beneficiaries would have modest gains—84 lives saved per year—but would not have a major impact on postoperative mortality for this population. Even in a scenario where both patients treated at 1- and 2-star hospitals (30.8% of patients) were relocated to 5-star hospitals, 208 lives among the 32 591 patients treated would be saved per year.

Together, these results suggest CMS Star Ratings are not particularly helpful in guiding patients, because star ratings may not account for a large degree of observed variation in postoperative deaths. To understand why, several factors should be considered. The star ratings are not specific to the surgical procedures performed; ratings are developed and applied at the hospital level and factors that go into ratings, although important, are unlikely to reflect the quality of care delivered for relatively uncommon procedures and are unlikely to be causal in the relationship between hospital and outcome. From the data presented, it is unclear how widely postoperative mortality ranged within each star group. Postoperative mortality rates will vary in these hospitals such that reliance on star ratings could lead a patient to move from a low-mortality 1-star hospital to a higher mortality 5-star hospital, and, in some jurisdictions, the best performing hospital may be a 3- or 4-star hospital. Because of how scores are generated, 5-star ratings may not equate perfectly with other hospital characteristics associated with better outcomes. Notably, CMS Star Ratings do not correlate with hospital volumes (3), a factor known to be strongly associated with surgical outcomes (5,6). Additionally, compared with lower rated hospitals, 5-star hospitals less commonly have intensive care units and larger hospitals less frequently receive 5-star ratings than smaller hospitals (7).

Despite the modest benefits at the population-averaged level, individual patients may find these results convincing enough to rely on CMS Star Ratings to select hospitals for their cancer care. Migration of patients (and associated revenue) from low-ranked hospitals may compel institutions to provide higher quality care. However, there are potential downsides to such a strategy for patients and the health-care system. Although this study identifies an association between star ratings and 90-day mortality, whether postoperative outcomes are better at the 5-star hospital closest to an individual patient than the closest lower ranked hospital will vary. Additionally, major patient movement may overwhelm higher ranked hospitals, leading to longer wait times with implications for long-term cancer outcomes not offset by reduced postoperative mortality. Previous work has shown that patients with less social support, lower income, and poorer health are less willing to travel to receive care (8), and Black patients more often receive care at low-quality, higher mortality hospitals even when they live closer to high-quality hospitals than White patients (9). Encouraging patients to select hospitals based on CMS Star Ratings could, therefore, widen existing disparities (10).

Though regionalization of cancer care makes practical sense for very high-risk procedures performed at low volumes, for more common procedures, greater gains may result not from bringing patients to high-quality institutions but from bringing elements of high-quality institutions to patients. Within institutions, the effects of regionalization on surgeon experience can potentially be re-created. Sahni et al. (11) have shown that operative mortality is related to a surgeon’s degree of specialization in a specific procedure (number of times the procedure was performed divided by the surgeon’s total operative volume) even after adjustment for procedure volume. Although individual surgeons may currently have low volumes of complex cancer surgeries, pooling referrals within hospitals and designating individuals to perform particular procedures can increase surgeon volumes. Additionally, previous studies have shown similar complication rates at high- and low-mortality hospitals (12,13), suggesting higher mortality arises from failure to rescue (FTR) patients who experience complications. High-volume hospitals may have lower mortality because of processes that lower FTR, such as closed intensive care units, overnight coverage, and dedicated rapid response teams (14). However, only a small degree of variation in FTR is accounted for by hospital characteristics and operative volume (15), so strategies beyond investment of resources should be considered. More generally, encouraging a safety culture and having escalation protocols can also lead to improvements in morbidity and mortality for surgical patients (16,17).

Improving the quality of care for patients requiring complex cancer surgery by redirecting patients to the highest performing hospital in their region, although a simple solution, will not be achieved by using the Overall Hospital Quality Star Ratings, and such an approach may increase existing disparities. Instead, applying elements of high-quality hospitals, particularly those that require minimal resource investment, can help bridge the quality gap.

## Notes

**Disclosures:** The authors have no conflicts of interest to disclose.

**Role of the authors:** FD drafted the manuscript. NNB provided critical review of the manuscript.
